# Study on the Fatigue and Healing Characteristics of Steel Slag Asphalt Concrete

**DOI:** 10.3390/ma18235361

**Published:** 2025-11-28

**Authors:** Heng Yuan, Haofeng Zheng, Hao Huang, Liantong Mo

**Affiliations:** State Key Laboratory of Silicate Materials for Architectures, Wuhan University of Technology, Wuhan 430070, China; 345049@whut.edu.cn (H.Y.); 345352@whut.edu.cn (H.Z.); 344828@whut.edu.cn (H.H.)

**Keywords:** steel slag, asphalt concrete, fatigue, healing

## Abstract

The fatigue healing mechanisms of steel slag asphalt concrete remain unclear and involve complex influencing factors. When used as an asphalt pavement material in actual road engineering projects, there is a risk of significant deviations in fatigue life predictions and insufficient stability in long-term service performance. In this study, traditional diabase asphalt concrete was used as a reference. Mix design was carried out for various steel slag asphalt mixtures, where steel slag coarse aggregates partially or entirely replaced diabase coarse aggregates. By using four-point bending fatigue testing, the fatigue life and stiffness modulus recovery capacity of steel slag asphalt concrete were analyzed after simulating low-temperature winter fatigue damage followed by healing at different temperatures (20 °C, 35 °C, 60 °C, and 75 °C). The test results indicated that the addition of steel slag coarse aggregates significantly affected the fatigue life and stiffness modulus of asphalt concrete. The use of coarser steel slag and autoclaved steel slag aggregates was beneficial for improving fatigue life. After experiencing low-temperature fatigue damage, increasing the healing temperature enhanced the modulus recovery effect but had a relatively low effect on life recovery. Overall, the stiffness modulus healing index of steel slag asphalt concrete exceeded 90%, while the fatigue life healing index ranged between 19% and 55%. After five fatigue healing cycles, the total fatigue life can be extended by 1.7 to 2.3 times. A life prediction model under multiple fatigue healing tests can be established using the stiffness modulus healing index and fatigue damage rate. Model predictions and measured results confirmed that the total fatigue healing life of asphalt concrete with the complete replacement of diabase coarse aggregates by steel slag coarse aggregates was greater than that of traditional diabase asphalt concrete.

## 1. Introduction

As the primary solid waste in the iron and steel industry, steel slag has a massive volume of discharge and stockpiling, making it imperative to vigorously pursue source reduction, resource utilization, and harmless disposal [1,2]. Because of its advantages such as high strength, great hardness, excellent wear resistance, abundant angularity, and strong adhesion to asphalt, steel slag aggregates can replace traditional anti-skid aggregates (e.g., diabase, basalt) in the surface wearing course of asphalt pavements [3,4,5,6,7]. Currently, steel slag aggregate is proved to be both environmentally and technically suitable for the application of anti-skid aggregates. It has been successfully used in numerous expressway projects, and thus relevant technical standards and application specifications have been established for steel slag asphalt concrete in China [8,9].

Steel slag asphalt concrete has been widely applied in some provinces such as Shanxi, Henan, and Shandong in China, primarily used as asphalt surface wearing courses. Currently, research on steel slag asphalt concrete mainly focuses on the issue of volume stability, while relatively few studies have been conducted on its fatigue healing behavior. Based on a fatigue test data analysis method using stiffness modulus and dissipated energy, Kavussi et al. [10] found that incorporating electric furnace steel slag can improve the fatigue life of asphalt concrete. Qazizadeh et al. [11] reported that asphalt mixtures with electric arc furnace and basic oxygen furnace slags incorporated exhibited higher fatigue life. Lan et al. [12] incorporated 9.5–16 mm steel slag coarse aggregates into asphalt concrete and found that when the steel slag content reached 50%, the fatigue life reached its maximum value while the initial stiffness modulus exhibited its minimum. Goli et al. [13] used steel slag coarse aggregates in warm mix asphalt mixtures, which enhanced the mixtures’ resistance to water damage and permanent deformation. Additionally, warm mix asphalt mixtures containing steel slag aggregates exhibited better fatigue performance than traditional hot mix asphalt mixtures.

The fatigue healing process of asphalt materials is a complex physical and chemical process, which is influenced by multiple factors such as type of asphalt, temperature, load interval time, damage degree, and aggregate characteristics. Chen et al. [14] investigated the self-healing performance of steel slag asphalt mixtures under microwave heating and found that healing temperature had the greatest impact on the healing rate, followed by the residual bending stiffness modulus, and the resting duration had the smallest impact. The optimal healing conditions were achieved at 80 °C with 60% residual bending stiffness modulus and 8 h of rest duration. Huang [15] carried out fatigue tests on nine types of modified asphalt mixtures and two types of base asphalt mixtures and found that SBS- and tire-rubber-modified asphalt mixtures showed higher fatigue and healing performance. Wang [16] found that asphalt type had the most significant influence on the healing ability of asphalt mixtures and other influencing factors were ranked by importance as follows: healing temperature, damage degree, healing time, and interval time. Xiao [17] used the four-point bending fatigue test to study the fatigue life of asphalt mixtures with different gradations under different strains, temperatures, initial damage rates, and healing times, and proposed a model that can accurately describe the fatigue life of asphalt mixtures. Xiang [18] found that healing temperature had the greatest impact on the fatigue life of asphalt mixtures, followed by asphalt binder type, loading strain, healing time, and damage degree. The healing indices of base asphalt and SBS-modified asphalt mixtures reached their maximum values at healing temperatures of 50 °C and 60 °C, respectively.

Currently, there are relatively few studies on the fatigue healing behavior of steel slag asphalt concrete and thus its mechanism remains unclear, with complex influencing factors. When steel slag asphalt concrete is used in actual road engineering, there are risks of large deviations in fatigue life prediction and insufficient stability in long-term service performance. In addition, the application of steel slag aggregates together with waste tire rubber powder can produce green, low-carbon, wear-resistant and anti-skid asphalt surface wearing course concrete, realizing the efficient resource utilization of waste rubber tires and steel slag solid waste [12,19,20]. According to the practical application of waste rubber-tire-modified asphalt and steel slag asphalt concrete in Henan province, this study carries out a series of laboratory tests on the fatigue and healing properties of steel slag asphalt concrete prepared using waste rubber tire plus SBS combined modified asphalt. The aim is focused on whether fatigue damage healing occurs after fatigue failure at low temperatures in winter, following the gradual temperature rise process in spring and summer.

For this purpose, traditional diabase asphalt concrete was used as a reference to design the mix proportion of steel slag asphalt mixtures where steel slag coarse aggregate replaces part or all of the diabase coarse aggregate. Multiple four-point bending fatigue tests were carried out to simulate the healing process at different temperatures after low-temperature fatigue failure in winter. According to the local monthly average temperature in spring and summer (March to August in Zhumadian, China), some representative healing temperatures were selected to reflect the actual pavement temperatures for the fatigue healing tests. The healing temperatures were designed to be gradually increased from 20 °C to 35 °C, 60 °C, and 75 °C, simulating the gradual temperature rise process of the asphalt pavement surface layer from spring to summer [21,22,23]. The temperature of 75 °C was selected to reflect the maximum surface temperature that the asphalt pavement surface layer could reach during summertime. The fatigue life and stiffness modulus recovery ability of steel slag asphalt concrete was analyzed according the fatigue data. Based on the cyclic fatigue healing test mode, the influence of self-healing on fatigue life extension and stiffness modulus recovery ability was quantified under different healing conditions. Finally, an evaluation method for the fatigue healing performance of steel slag asphalt concrete was proposed, and a fatigue life prediction model for multiple fatigue healing tests was established.

## 2. Materials and Methods

### 2.1. Materials

The asphalt binder used in this study was crumb rubber plus SBS combined modified asphalt, supplied by the Shang-luo Expressway project in Henan Province. This asphalt binder had a density of 1.043 g/cm^3^, a penetration of 46, a softening point of 75.5 °C, and a ductility of 23 cm. The steel slag coarse aggregates consisted of two size fractions: 4.75–9.5 mm and 9.5–16 mm. These steel slag aggregates were produced by Xinyang Iron and Steel Co., Ltd. (Xinyang, China) using steam-treated steel slag that was crushed, stockpiled, and aged for no less than six months. The steel slag had a free calcium oxide content of 1.9% and a water immersion expansion rate of 1.4%. The used steel slag coarse aggregate had an apparent relative density of 3.332, a water absorption rate of 1.7%, a crushing value of 18.6%, a Los Angeles abrasion loss of 18.2% and a polished stone value of 48. All its properties met the design specification requirements [9], making it suitable for asphalt pavement construction. The diabase coarse aggregates comprised three size fractions: 2.36–4.75 mm, 4.75–9.5 mm, and 9.5–16 mm. The used diabase coarse aggregate had an apparent relative density of 2.949, a water absorption rate of 0.6%, a crushing value of 10.6%, a Los Angeles abrasion loss of 12.2% and a polished stone value of 47. Fine aggregates smaller than 2.36 mm were limestone manufactured sand, and the filler was limestone mineral powder.

### 2.2. Asphalt Mixture Proportion Design

The rubber-powder-composite-modified asphalt mixture with WRAC-13 gradation was used for the mix design of steel slag asphalt concrete. During the mix design, diabase aggregates were used as the benchmark to conduct an experimental study on replacing diabase coarse aggregates with steel slag coarse aggregates. The upper and lower limits of WRAC-13 gradation are shown in Table 1. The percentages of diabase coarse aggregate, steel slag coarse aggregate, limestone fine aggregate and filler were specially design based on the grading of these raw materials as mentioned above to achieve a similar combined aggregate gradation as listed in Table 1. To ensure the gradation consistency, the combined aggregate gradations of these five mix proportions took the passing percentage of 4.75 mm, 2.36 mm and 0.075 mm as the key control sieves. For various WRAC-13 gradations, the passing percentage of 4.75 mm was controlled at around 29%, 2.36 mm at around 22%, and 0.075 mm at around 6%, respectively. Their combined aggregate gradations are shown in Table 1.

The asphalt mixture mix design was carried out in accordance with JTG F40-2004 Technical Specifications for Construction of Highway Asphalt Pavements [24]. Tests were conducted under various asphalt–aggregate ratios (e.g., 5.1%, 5.4%, 5.7%, 6.0%, 6.3%). Combined with Marshall stability, flow value, air voids, voids in mineral aggregate and voids filled with asphalt, the optimal asphalt–aggregate ratio was determined with target air voids around 4%.

The mix proportions of different asphalt mixtures are shown in Table 2. In this study, the optimal asphalt–aggregate ratio for all types of asphalt concrete was determined as 5.7% according to Marshall testing with target air voids around 4%. In the above mix proportion design, Mix proportion A consisted of diabase coarse aggregate entirely to prepare asphalt concrete. Mix proportions B and C used steel slag coarse aggregates of the same particle size (stockpiled on-site) to replace the 9.5–16 mm and 4.75–9.5 mm diabase coarse aggregates in Mix proportion A, respectively. Mix proportion D used steel slag coarse aggregates of the same particle size to replace both the 4.75–9.5 mm and 9.5–16 mm diabase coarse aggregates in Mix Proportion A. Mix proportions B, C, and D contained 1 to 2 fractions of steel slag coarse aggregates, thus having potential volume stability issues. To facilitate the comparative analysis of the influence of stabilized steel slag coarse aggregate, Mix proportion E consisted of steel slag coarse aggregates treated by autoclaving (9 h, 0.16 MPa) in the laboratory to prepare asphalt concrete, which allows for a comparative analysis with Mix proportion D on the impact of steel slag particles with volume instability.

### 2.3. Test Methods

In this study, a UTM-130 Universal Testing Machine (IPC Global Pty Ltd., Melbourne, Australia) was used to conduct four-point bending fatigue tests. Prior to the tests, asphalt mixture slabs with dimensions of length of 400 mm, width of 300 mm and thickness of 75 mm were compacted according to the five mix proportions listed in Table 1. These slabs were then saw into beam specimens with dimensions of length of 380 mm ± 5 mm, height of 50 mm ± 6 mm, and width of 63 mm ± 6 mm.

**Figure 1:**
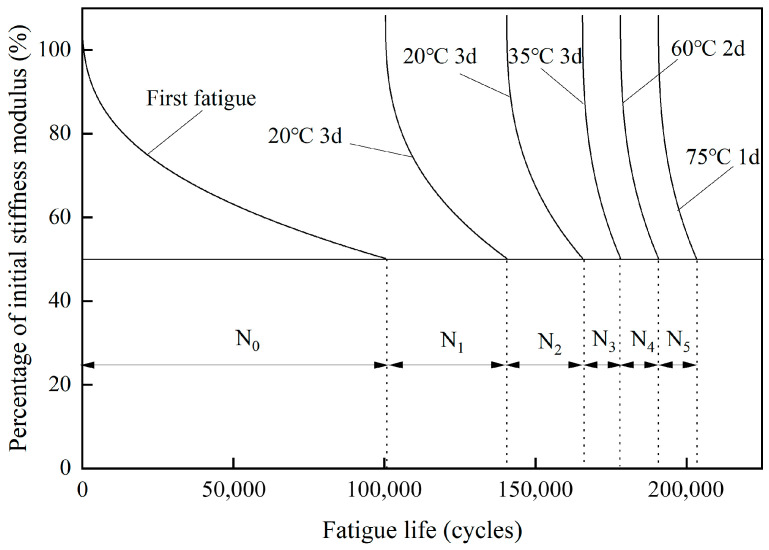
Test method of fatigue–healing–fatigue testing.

To obtain insight into whether fatigue damage healing occurs in steel slag asphalt concrete after fatigue failure at low temperatures in winter, followed by the gradual temperature rise in spring and summer, each beam specimen was subjected to six individual fatigue tests (as shown in Figure 1). The first test was a direct fatigue test, while the remaining five tests were multiple fatigue tests under different healing conditions. Detailed information on these fatigue tests was given as follows:

First Test: To simulate the winter low-temperature fatigue damage process of asphalt concrete, the first fatigue test was conducted at 0 °C under strain control mode, with a loading frequency of 10 Hz and a strain level of 400 με. The fatigue test was stopped when the stiffness modulus dropped to 50% of the initial stiffness modulus.

Second Test: After the first fatigue test, the beam specimen was carefully removed and placed in a 20 °C incubator for 3 days to allow internal fatigue damage to heal. After the healing period, the specimen was reinstalled in the UTM-130 system for the four-point bending fatigue test under the same conditions as the first fatigue test (0 °C, 10 Hz loading frequency, 400 με strain level). The test was stopped again when the stiffness modulus decreased to 50% of the initial modulus from the first fatigue test. This fatigue test aimed to verify the healing capacity after the first low-temperature fatigue failure following a 3-day incubation at 20 °C (simulating temperature rise from 10 °C to 20 °C).

Third Test: After the second fatigue test, the beam specimen was carefully removed, and the procedure of the second fatigue test was repeated. This aimed to verify the healing and recovery capacity after the second fatigue failure, again with a 3-day incubation at 20 °C.

Fourth Test: After the third fatigue test, the beam specimen was carefully removed and placed in a 35 °C incubator for 3 days for healing. Subsequent four-point bending fatigue tests were conducted under the same conditions as before. The fatigue test was stopped when the stiffness modulus dropped to 50% of the initial modulus from the first test. This fatigue test aimed to examine the fatigue damage recovery capacity after the third fatigue failure, with the healing temperature increasing from 20 °C to 35 °C for 3 days.

Fifth Test: After the fourth fatigue test, the beam specimen was carefully removed and placed in a 60 °C incubator for 2 days for healing. Subsequent four-point bending fatigue tests were carried out to verify the recovery capacity after the fourth fatigue failure, with the healing temperature increased from 35 °C to 60 °C for 2 days.

Sixth Test: After the fifth fatigue test, the beam specimen was carefully removed and placed in a 75 °C incubator for 1 day for healing. Subsequent four-point bending fatigue tests were conducted to examine the recovery capacity after the fifth fatigue failure when the healing temperature was increased from 60 °C to 75 °C for 1 day, simulating the maximum temperature asphalt pavements reach in summer to assess its effect on fatigue damage recovery.

According to the above fatigue–healing–fatigue test method, the curves of asphalt concrete stiffness modulus varying with the number of cyclic loads were obtained as shown in Figure 1. In Figure 1, N_0_ represents the fatigue life from the first fatigue test, while N_1_, N_2_, N_3_, N_4_ and N_5_ denote the fatigue lives obtained from the five successive healing fatigue tests. The healing temperature was gradually increased from 20 °C to 35 °C, 60 °C, and 75 °C, simulating the gradual temperature rise process of asphalt pavement surface layer from winter to spring and summer. A longer healing time (3 days) was adopted when the healing temperature was 20 °C and 35 °C; however, as the healing temperature rose to 60 °C and 75 °C, the healing time was reduced accordingly to 2 days and 1 day, respectively.

In order to explore the recovery degree of the fatigue performance of steel slag asphalt concrete after winter fatigue and healing during the coming spring and summer, two healing indices based on fatigue life and stiffness modulus were proposed. The fatigue life healing index (HI) was defined using Equation (1):

(1) $${HI}_{i}=\frac{N_{fi}}{N_{f0}}\times 100\%$$

where HI_i_ is the fatigue life healing index of asphalt concrete in the i-th fatigue healing test; N_fi_ is the fatigue life of asphalt concrete in the i-th fatigue healing test; and N_f0_ is the fatigue life of asphalt concrete in the first fatigue test.

Similarly, the stiffness modulus recovery degree can be used to evaluate the healing performance of asphalt concrete, and the stiffness modulus healing index (HE) was introduced, as shown in Equation (2):

(2) $${HE}_{i}=\frac{E_{0i}}{E_{0}}\times 100\%$$

where HE_i_ is the initial modulus healing index of asphalt concrete in the i-th fatigue healing test; E_0i_ is the initial modulus of asphalt concrete in the i-th fatigue healing test; E_0_ is the initial modulus of asphalt concrete in the first fatigue test.

## 3. Results

### 3.1. Fatigue Performance Analysis

In order to compare the influence of the addition of steel slag coarse aggregates on the fatigue performance of asphalt concrete, four-point bending fatigue tests were conducted on asphalt concrete where diabase aggregates were replaced by steel slag aggregates of different particle sizes. The obtained test results are shown in Table 3. Compared with the fatigue life of diabase asphalt concrete (Mix proportion A), Mix proportions B, E, and D contained 33.2%, 70.5%, and 70.5% steel slag coarse aggregates, respectively, with their fatigue lives increasing by 84.5%, 44.3%, and 4.1% correspondingly. However, Mix proportion C, which had a steel slag content of 36.4%, showed a decrease in fatigue life by 26.5% instead of an increase. The fatigue lives of different asphalt concretes were ranked as follows: Mix proportion B > Mix proportion E > Mix proportion D > Mix proportion A > Mix proportion C.

Compared with Mix proportion A without steel slag, Mix proportion B (incorporating only 9.5–16 mm steel slag) exhibited a significant improvement in fatigue life, while Mix proportion C (incorporating only 4.75–9.5 mm steel slag) showed a slight decrease. Mix proportion D (incorporating both 4.75–9.5 mm and 9.5–16 mm steel slag) had a marginal increase in fatigue life, whereas Mix proportion E (incorporating 4.75–9.5 mm and 9.5–16 mm steel slag subjected to autoclaving treatment) demonstrated a notable improvement. It can be seen that steel slag aggregates of different particle sizes had varying effects on the fatigue life of asphalt concrete. In general, 4.75–9.5 mm steel slag aggregates exerted a negative impact, while 9.5–16 mm steel slag aggregates significantly enhanced the fatigue life. Additionally, the fatigue performance was also notably improved by using steel slag aggregates with autoclaving treatment.

Compared with the initial stiffness modulus of Mix proportion A (diabase asphalt concrete), the initial stiffness moduli of Mix proportions B, C, D, and E decreased by 14.3%, 4.0%, 9.6%, and 6.5%, respectively. The initial stiffness moduli of different asphalt concretes were ranked as: Mix proportion A > Mix proportion C > Mix proportion E > Mix proportion D > Mix proportion B. Mix proportions A and C had higher initial stiffness moduli but lower fatigue lives. A larger initial stiffness modulus caused asphalt concrete to bear greater stress and suffer more fatigue damage when subjected to the same strain level. Under strain control mode, steel slag asphalt concrete with a smaller initial stiffness modulus tended to exhibit better fatigue resistance.

**Figure 2:**
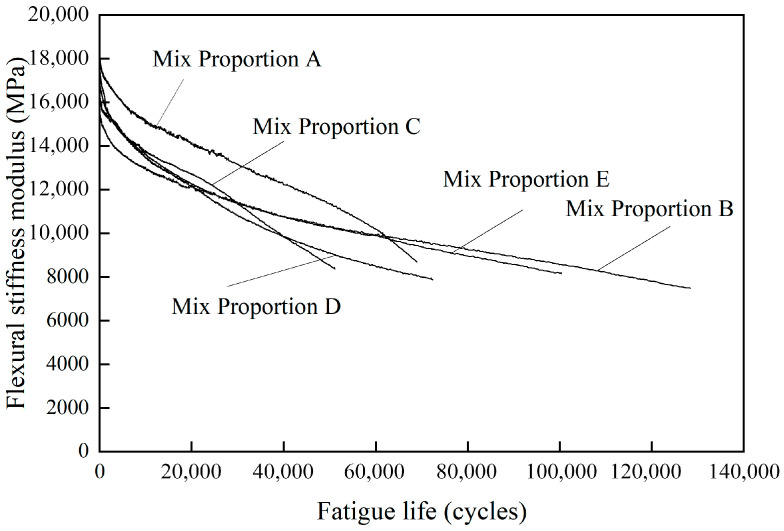
Fatigue curves of different asphalt concrete.

Figure 2 shows the curves of stiffness modulus for different asphalt concretes under fatigue loading. With the increase in the number of loading cycles, the stiffness modulus of asphalt concrete gradually decreased. The reduction in the stiffness modulus of asphalt concrete with increasing cyclic loads exhibited three stages of change [25]: the first stage was the initial inflection point region, where the stiffness modulus dropped rapidly; the second stage was the steady decline stage, during which the stiffness modulus decreased approximately linearly; the third stage was the crack propagation stage, where the stiffness modulus dropped rapidly until the specimen fractures.

Mix proportions A and C showed the third stage during the fatigue test and the specimens failed earlier, indicating that the fatigue performance of Mix proportions A and C is poor. In contrast, the stiffness modulus curves of Mix proportions B, D, and E did not show the third stage, and they exhibited longer fatigue lives.

### 3.2. Healing Performance Analysis

#### 3.2.1. Fatigue Results of Different Healing Condition

In winter, asphalt concrete is prone to fatigue damage due to low-temperature environments and frequent traffic loads. The fatigue damage can be characterized by the propagation of microcracks on the surface, changes in the material’s microstructure, and a decline in overall performance. However, with the arrival of spring and summer, the temperature gradually rises, and this process provides favorable conditions for the performance recovery of asphalt concrete because of the self-healing effect. In spring and summer, as the temperature increases, the asphalt binder in asphalt concrete begins to soften and its viscoelasticity enhances, which helps fill and heal the microcracks caused by fatigue. At the same time, the adhesion between asphalt and aggregates is also improved, further enhancing the overall stability and durability of asphalt concrete.

**Figure 3:**
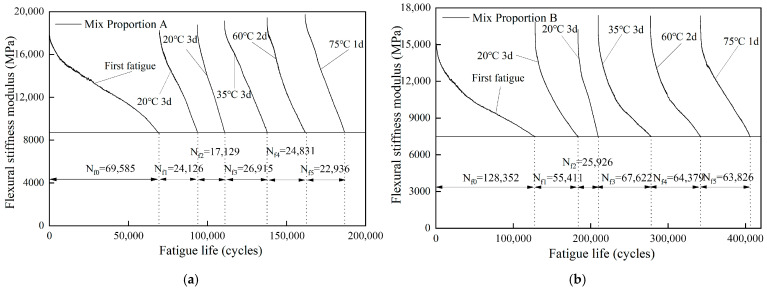

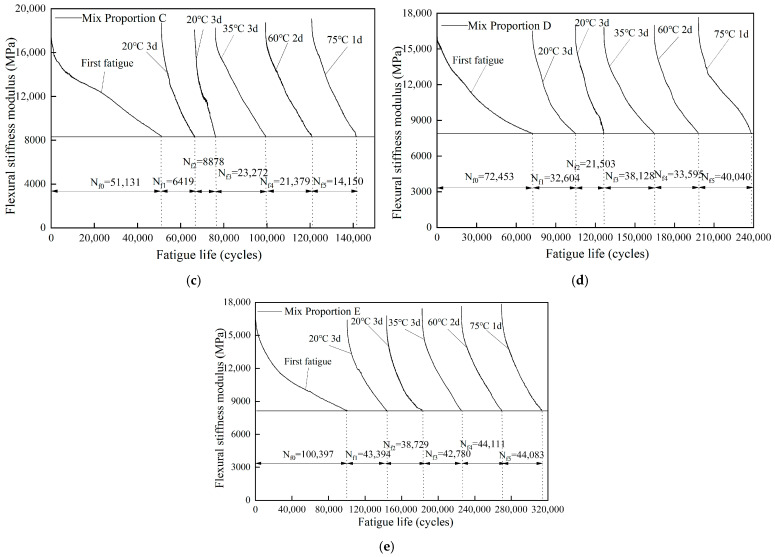
Fatigue curves of different asphalt concrete after five healing periods.

Figure 3 presents the stiffness modulus curves of different steel slag asphalt concretes in the first fatigue test and the five fatigue healing tests. It can be seen that the fatigue performance of asphalt concrete was unable to be fully restored after healing, and different asphalt concretes exhibited significant differences in healing performance under the same healing conditions. Healing durations at different temperatures can result in a certain life extension and almost a full modulus recovery.

#### 3.2.2. Effect of Different Healing Conditions on Initial Stiffness Modulus

**Figure 4:**
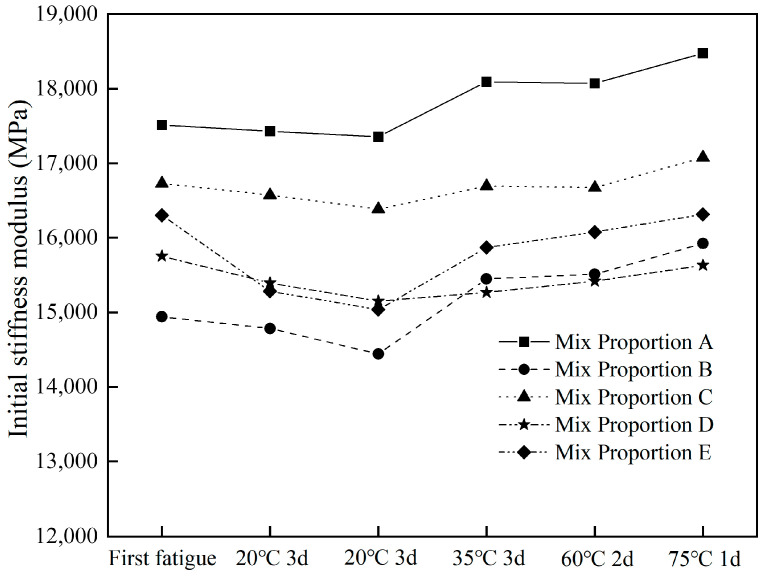
Changes in the initial modulus for different asphalt concretes.

The changes in the initial stiffness moduli of different asphalt concretes after fatigue healing are shown in Figure 4. As observed in Figure 4, the initial stiffness moduli of different asphalt concretes changed slightly after different healing cycles. In the first two healing–fatigue tests, the initial stiffness modulus of asphalt concrete showed a downward trend. This was because the low healing temperature was insufficient to repair the microcracks generated during the fatigue damage process, leading to a decrease in stiffness modulus. In the last two fatigue healing tests, the initial stiffness modulus of asphalt concrete showed an upward trend. When the healing temperature was increased to 60 °C and 75 °C, it exhibited excellent healing effects, promoting the repair of microcracks in asphalt concrete. It should be noted that heating at 60–75 °C for days can cause binder oxidation and thus increase the stiffness and affect the healing performance of asphalt concrete [18].

The initial stiffness modulus of diabase asphalt concrete (Mix proportion A) remained the highest throughout all the tests, which was due to the addition of steel slag reducing the stiffness modulus of asphalt concrete. Based on the average initial stiffness modulus of different asphalt concretes in these six fatigue tests, the influence of steel slag incorporation was ranked as follows: Mix proportion A (17,823.1 MPa) > Mix proportion C (16,688.7 MPa) > Mix proportion E (15,813.9 MPa) > Mix proportion D (15,436.0 MPa) > Mix proportion B (15,175.8 MPa).

Compared with the average initial stiffness modulus of Mix proportion A (diabase asphalt concrete), Mix proportions C, E, D, and B incorporated 36.4%, 70.5%, 70.5%, and 33.2% steel slag, respectively, and their average initial stiffness moduli decreased by 6.36%, 11.27%, 13.39%, and 14.85% accordingly. It can be concluded that the addition of steel slag aggregates reduced the initial stiffness modulus of asphalt concrete. In general, 9.5–16 mm steel slag aggregates had a greater impact, reducing the average initial stiffness modulus by 14.85%; 4.75–9.5 mm steel slag aggregates had a smaller impact, decreasing the average initial stiffness modulus by 6.36%; while Mix proportions E and D, which incorporate both 4.75–9.5 mm and 9.5–16 mm steel slag aggregates, had a moderate impact.

**Figure 5:**
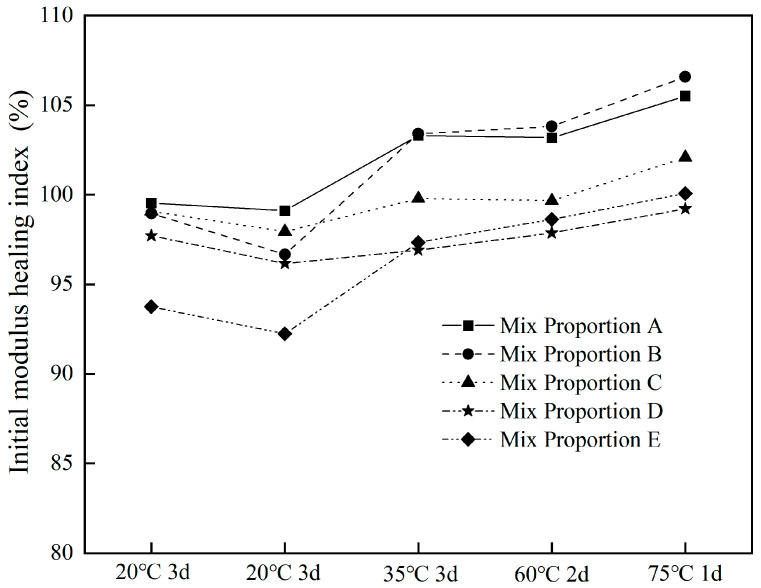
Initial modulus healing index for different asphalt concretes.

Figure 5 presents the stiffness modulus healing indices (HE) of different asphalt concretes. The stiffness modulus healing index showed a downward trend in the first two fatigue tests after healing at 20 °C for 3 days; however, as the temperature gradually increased thereafter, the stiffness modulus healing index continuously improved, basically approaching 100%, or even exceeding 100%. After healing at 20 °C for 3 days, the initial stiffness modulus could recover well, with the healing index ranging from 93.7% to 99.5%; while after healing at 75 °C for 1 day, the healing index ranged from 99.2% to 106.6%. Overall, the stiffness modulus healing index did not change significantly, fluctuating within the range of 92.2% to 106.6%. Taking Mix proportion A as an example, its stiffness modulus healing indices in the five fatigue healing tests were 99.5%, 99.1%, 103.3%, 103.2%, and 105.5% in sequence, showing minimal overall variation. The test results indicated that after low-temperature fatigue damage, the stiffness moduli of different asphalt concretes can basically recover to the initial level through healing under temperature rise after long healing time. Mullapudi [26] found that there was no significant difference between the healing abilities of the asphalt mixes healed at 40 °C and 60 °C by using indirect tensile fatigue test. The healing indices strongly depends on the damage level. The modulus healing index can reach around 73% at a damage level of 50%. Li [27] reported that asphalt mixtures containing the optimal 2% iron slag can recover about 59% of their original structural strength under the optimal healing temperature of 60 °C and a damage degree of 30%. Zhang [28] reported that asphalt mixtures with 80% steel slag content exhibited a healing index of 92.8% in strength recovery using semi-circular specimens under microwave healing. By comparing with other studies, it can be seen that steel slag asphalt concrete had good healing performance.

When using the average value of the stiffness modulus healing indices from the five fatigue healing tests to comprehensively evaluate the healing performance of different asphalt concretes, the ranking was found to be as follows: Mix proportion A (102.1%) > Mix proportion B (101.9%) > Mix proportion C (99.7%) > Mix proportion D (97.6%) > Mix proportion E (96.4%). Compared with Mix proportion A (diabase asphalt concrete), Mix proportions B, C, D, and E, which incorporated 33.2%, 36.4%, 70.5%, and 70.5% steel slag, respectively, showed reductions of 0.24%, 2.37%, 4.46% and 5.6% on the stiffness modulus healing indices accordingly. It can be seen that the addition of steel slag had a negative impact on the recovery of the initial modulus of asphalt concrete, but this impact was relatively very small.

#### 3.2.3. Effect of Healing Conditions on Fatigue Life

**Figure 6:**
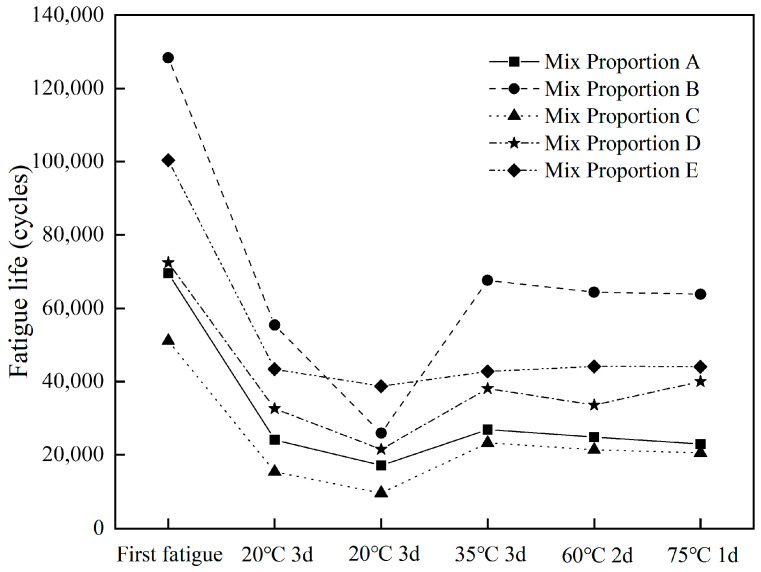
Fatigue life changes with different asphalt concretes.

The changes in fatigue lives of different asphalt concretes after fatigue healing tests are shown in Figure 6. In the first two healing fatigue tests, the fatigue lives of different asphalt concretes showed a downward trend, which is because the healing temperature of 20 °C was relatively low to repair fatigue damage. In the third fatigue healing test, the fatigue lives of different asphalt concretes increased significantly, indicating that a healing temperature of 35 °C can effectively promote the healing of fatigue damage in asphalt concrete. However, in the last two fatigue tests, the fatigue life changed slightly, which may be due to the small influence of temperature on the healing performance of asphalt concrete within the range of 35 °C to 75 °C.

The total fatigue lives of different asphalt concretes before and after healing was ranked as follows: Mix proportion B (405,516 cycles) > Mix proportion E (313,449 cycles) > Mix proportion D (238,323 cycles) > Mix proportion A (185,522 cycles) > Mix proportion C (141,311 cycles). This overall ranking was consistent with the results of the first fatigue test.

**Figure 7:**
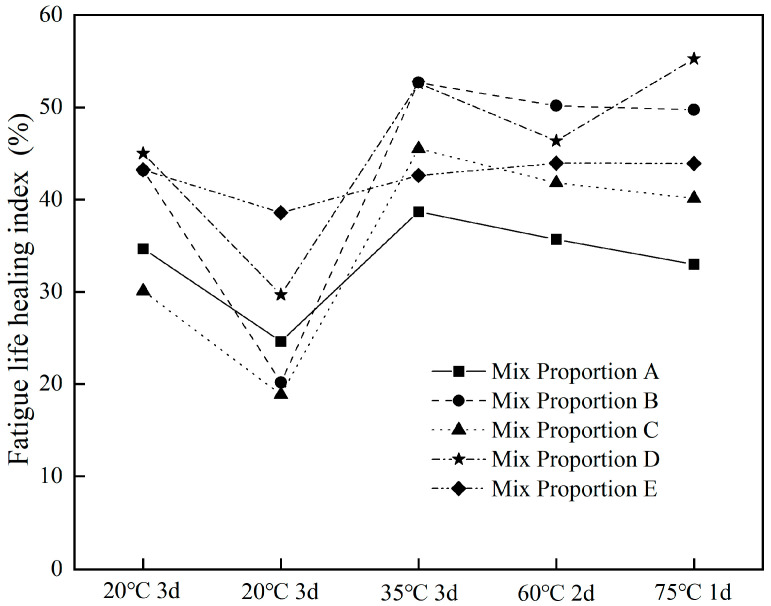
Fatigue life healing index with different asphalt concretes.

Figure 7 shows the fatigue life healing indices of different asphalt concretes. After healing at 20 °C for 3 days, the fatigue lives of different asphalt concretes could recover to 30–45% of the original; however, after the second healing at 20 °C for 3 days, it could only recover to 18.9–38.6% of the original. When the healing temperature was further increased to 35 °C, the fatigue lives of all asphalt concretes increased significantly, recovering to 38.7–52.7% of the original. After healing at 60 °C for 2 days, the fatigue lives of different asphalt concretes changed slightly, recovering to 33.0–55.3% of the original. After healing at 75 °C for 1 day, the fatigue lives of different asphalt concretes could recover to 35.7–50.1% of the original. The fatigue life healing indices of different asphalt concretes changed slightly after the last two healing periods at 60 °C and 75 °C. Xiang [18] reported that the healing indices of unmodified and SBS-modified asphalt mixtures reached their maximum values at healing temperatures of 50 °C and 60 °C, respectively, by four-point bending fatigue test. The fatigue life healing index can reach 65~85% after healing for 50 h. Mullapudi [26] found that there was no significant difference between the healing abilities of the asphalt mixes healed at 40 °C and 60 °C by using indirect tensile fatigue test. The healing indices strongly depend on the damage level. The fatigue life healing index can reach around 70% at a damage level of 50%. Chen [14] reported that the optimal healing conditions of steel slag asphalt mixture under microwaves were achieved at 80 °C with 60% residual bending stiffness modulus and 8 h of rest duration, the fatigue life healing index was about 75%. Liu [29] reported that the self-healing rate of steel slag asphalt mixture can be increased by more than 30% under microwave heating. By comparing with other studies, it can be seen that steel slag asphalt concrete had a healing performance similar to other mixtures in terms of life extension.

After five healing periods, the total life extension ratios of different asphalt concretes were: Mix proportion D (228.9%) > Mix proportion B (215.9%) > Mix proportion E (212.3%) > Mix proportion C (176.4%) > Mix proportion A (166.6%). The fatigue life extension ratios of asphalt concretes with steel slag were all better than those without steel slag.

Overall, in the first three fatigue healing tests, the fatigue life healing index showed a trend of first decreasing and then increasing, while in the last two fatigue healing tests, it changed slightly. In the first three fatigue tests, since the healing conditions remained 20 °C in the second fatigue test, the fatigue performance of asphalt concrete was difficult to recover; however, when the healing temperature was later increased to 35 °C, the fatigue performance of asphalt concrete improved significantly. From the results of the last three fatigue tests, the fatigue life healing indices of asphalt concretes with different mix proportions changed slightly. This was because after the healing temperature reached a certain level, increasing the temperature further had little effect on the recovery of the fatigue performance of asphalt concrete. It should be noted that healing at high temperature could result in the aging of asphalt binder. This may lead to the hardening and decreased fluidity of the asphalt binder, thus reducing crack healing ability. Therefore, the healing effect of asphalt mixture showed a trend of increasing first and then decreasing with the rise in healing temperature, which has been confirmed by other studies [18,26,27]. Tang [30] et al. reported that the optimal healing temperature of asphalt binder was close to its softening point temperature.

Among all of the asphalt concretes, the fatigue life healing indices of Mix proportions A and C were consistently low under different conditions. This was the reason that the degree of fatigue damage was relatively high during the first fatigue test, resulting in severe internal structure damage. In general, the healing performance of asphalt concrete improved after incorporating steel slag coarse aggregates.

#### 3.2.4. Effect of Healing Conditions on Fatigue Damage Rate

**Figure 8:**
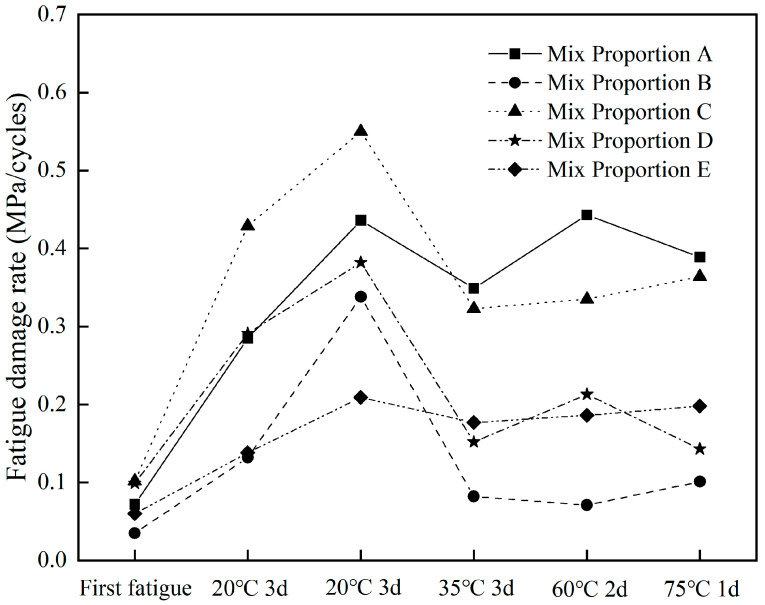
Fatigue damage rate in the second stage for different asphalt concretes.

To further analyze the fatigue healing performance of different asphalt concretes, the stiffness modulus decay rate k in the second steady stage of the fatigue curve was used as the fatigue damage rate of asphalt concrete. Figure 8 shows the variation in the fatigue damage rate for different asphalt concretes. The fatigue damage rate increased in the first two fatigue tests after healing, while it showed little change in the latter two fatigue tests after healing. Overall, the order of k values was as follows: Mix proportion C > Mix proportion A > Mix proportion D > Mix proportion E > Mix proportion B. As can be seen from Figure 8, the fatigue damage rates of Mix proportions C and A were consistently high, while those of Mix proportions B, D, and E were consistently low. This was consistent with the previous fatigue life results, indicating that the k value can reflect the fatigue resistance of asphalt concrete to a certain extent.

Under the strain-controlled mode, the fatigue damage process of the asphalt mixture is usually divided into three stages [25]. The second stage can be considered as the linear damage stage, where the stiffness modulus shows a linear downward trend with the increase in the number of fatigue cycles and accounts for most of the entire fatigue process. Moreover, this stage is widely used to predict the fatigue life of asphalt mixture for its simplicity. If the second stage lasts long enough to eliminate the effects of the first and third stage, this method can yield a good prediction result that is close to the actual measured fatigue life. For example, Qu et al. [31] accurately predicted the fatigue healing life of asphalt mixture using the linear fatigue damage accumulation rule. Since fatigue life is characterized by the number of loading cycles when the stiffness modulus decreases to 50% of the initial modulus, the total fatigue life under different healing conditions can be predicted using the initial modulus of each fatigue test of asphalt concrete and the stiffness modulus decay rate k:

(3) $$N_{P}=\frac{E_{0}\times 50\%}{k_{0}}+\frac{E_{01}-E_{0}\times 50\%}{k_{1}}+\frac{E_{02}-E_{0}\times 50\%}{k_{2}}+\frac{E_{03}-E_{0}\times 50\%}{k_{3}}+\frac{E_{04}-E_{0}\times 50\%}{k_{4}}+\frac{E_{05}-E_{0}\times 50\%}{k_{5}}$$

where k_0_ is the stiffness modulus decay rate of the asphalt concrete in the first fatigue test; k_i_ is the stiffness modulus decay rate of the asphalt concrete after the i-th healing; E_01_, E_02_, E_03_, E_04_, E_05_ are the initial moduli of the fatigue tests from the first to the fifth healing.

The initial modulus after healing can be characterized by multiplying the initial modulus of the first fatigue test of asphalt concrete by the modulus healing index. By combining Equations (2) and (3), Equation (4) is obtained.

(4) $$N_{P}=E_{0}\times (50\%\frac{1}{k_{0}}+\sum_{i=1}^{5}\frac{{HE}_{i}-50\%}{k_{i}})$$

It can be seen from Equation (4) that the total fatigue life of asphalt concrete before and after healing consists of two parts, which represent the fatigue life of asphalt concrete before healing and the extended life after healing, respectively.

As shown in Figure 5, the variation range of the stiffness modulus healing index HE_i_ of different asphalt concretes in all fatigue tests was ranged from 92.2% to 106.6%. Therefore, the approximate value HE_i_ around 100% can be taken, and Equation (4) can be further simplified as:

(5) $$N_{P}=E_{0}\times 50\%\sum_{i=0}^{5}\frac{1}{k_{i}}$$

It can be seen that the total fatigue life was related to the initial modulus of the first fatigue test and the stiffness modulus decay rate k of each fatigue test. The fatigue life prediction results of asphalt concretes with different mix ratios are shown in Table 4.

**Figure 9:**
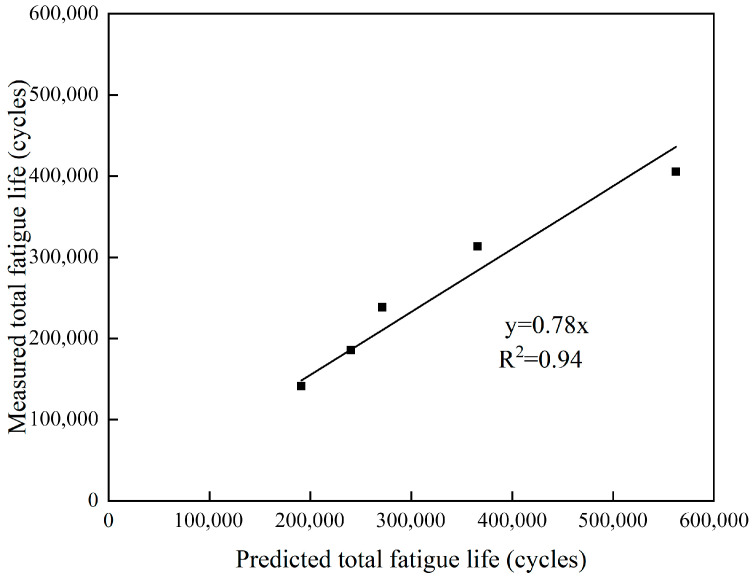
Comparison between the predicted total fatigue life and measured total fatigue life after healing.

The predicted values of total fatigue life for different mix ratios calculated by Equation (5) were compared with the measured total fatigue life, and the results are shown in Figure 9. It can be seen from Figure 9 that there was a good linear relationship between the total predicted fatigue life and the measured total fatigue life. In general, the life prediction based on Equation (5) using the second-stage slope resulted in an overestimation of fatigue life, as indicated in Table 4. The predicting fatigue life deviation ranged from 13.8% to 38.7%. It seemed that the fatigue life prediction based on linear damage accumulation together with the 50% modulus failure criterion was simple and practical. However, it lost some accuracy, which depends on whether the second stage is long enough or not to reduce the effects of the first and third stages. In this study, the fatigue test was carried out at 0 °C under strain control mode with a strain level of 400 με. This led to a relatively low number cycle to fatigue failure based on 50% modulus failure criterion. As a result, the life prediction based on the second-stage slope tended to be longer than the actual measured fatigue life.

By further introducing a correction coefficient of 0.78, a fatigue healing life prediction model based on the initial modulus and the stiffness modulus decay rate can be established. From the measured and predicted results of total fatigue life in Table 4, it can be seen that Mix proportion B, which only incorporates particles with a size of 9.5–16 mm, had the highest total fatigue life. Mix proportion C, which only incorporates particles with a size of 4.75–9.5 mm, had a slightly lower total fatigue life than the diabase asphalt mixture. However, Mix proportions D and E, in which all diabase coarse aggregates were replaced by steel slag coarse aggregates, had significantly improved total fatigue life. Therefore, the model prediction and measured results confirmed that the total fatigue life of asphalt concrete in which all diabase coarse aggregates were replaced by steel slag coarse aggregates was greater than that of traditional diabase asphalt concrete.

## 4. Conclusions

A series of four-point bending fatigue tests were conducted to study the fatigue healing performance of asphalt concrete in which part or all of diabase coarse aggregates were replaced by steel slag coarse aggregates. The fatigue life and stiffness modulus recovery ability of steel slag asphalt concrete after low-temperature fatigue damage and subsequent healing at 20 °C, 35 °C, 60 °C and 75 °C were analyzed, and the main conclusions are as follows:

(1) The incorporation of steel slag had a significant impact on the fatigue life and stiffness modulus of asphalt concrete. The steel slag content or particle size should be optimized based on the fatigue results of traditional asphalt concrete as a reference to ensure the lower limit of fatigue life. The use of steel slag with a coarse particle size of 9.5–16 mm and autoclaving treated steel slag was beneficial to improve the fatigue life of steel slag asphalt concrete.
(2) After steel slag asphalt concrete had undergone low-temperature fatigue damage, increasing the healing temperature had a good effect on modulus recovery, but its effect on life recovery was relatively limited. Overall, the stiffness modulus healing index of steel slag asphalt concrete was greater than 90%, while the life healing index ranged from 19% to 55%. After five fatigue healing tests at different temperatures, its total life can be extended by 1.7 to 2.3 times.
(3) Multiple fatigue healing tests confirmed that the fatigue life of steel slag asphalt concrete can be effectively extended after winter low-temperature fatigue damage was healed by the temperature rise in spring and summer. A fatigue healing life prediction model can be established through the initial modulus and the stiffness modulus decay rate under different healing conditions. The model prediction and measured results confirmed that the fatigue healing performance of asphalt concrete in which all diabase coarse aggregates were replaced by steel slag coarse aggregates was superior to that of traditional diabase asphalt concrete.

This study was limited to only one type of asphalt binder, one type of steel slag, and one type of mineral aggregate. Furthermore, only one mixture gradation and one fatigue strain level was considered. More tests are thus needed to better understand the fatigue and healing behavior of steel slag asphalt concrete due to its uncertainty and large variability. Heating at 60–75 °C for several days may cause binder oxidation and the effect on healing should be further investigated. The proposed fatigue healing life prediction model also needs further validation.

## Figures and Tables

**Table 1:** Combined aggregate gradation of WRAC-13 asphalt mixture.

Type of Gradation	Passing Percentage at Different Sieve Sizes									
	16	13.2	9.5	4.75	2.36	1.18	0.6	0.3	0.15	0.075
Upper limit	100	100	75	39	30	22	18	14	11	8
Lower limit	100	80	62	25	18	14	8	6	5	5
Mix proportion A	100	94.6	68.7	29.7	22.2	17.7	13.9	11.2	8.3	6.1
Mix proportion B	100	94.6	66.1	29.1	23.0	18.3	14.4	11.5	8.5	6.2
Mix proportion C	100	97.4	65.5	29.7	22.2	17.7	14.0	11.2	8.4	6.2
Mix proportion D	100	97.7	66.6	29.3	23.1	18.3	14.4	11.5	8.5	6.2
Mix proportion E	100	94.6	68.7	29.7	22.2	17.7	13.9	11.2	8.3	6.1

**Table 2:** Mix design of different WRAC-13 asphalt mixtures.

Type of Aggregate	Aggregate Size (mm)	Mix Proportion (%)				
		A	B	C	D	E
Steel slag	9.5–16	-	33.2	-	31.0	31.0
Steel slag	4.75–9.5	-	-	36.4	39.5	39.5
Diabase	9.5–16	31.0	-	33.2	-	-
Diabase	4.75–9.5	34.0	36.4	-	-	-
Diabase	2.36–4.75	7.0	6.6	6.6	6.2	6.2
Limestone	0–2.36	22.0	18.1	18.1	17.6	17.6
Filler	-	6.0	5.7	5.7	5.7	5.7
Asphalt–aggregate ratio	-	5.7	5.7	5.7	5.7	5.7

**Table 3:** Fatigue test results of different steel slag asphalt concretes.

Type of Mix	Initial Stiffness Modulus (MPa)	Fatigue Life (Cycles)
A	17,430.8	69,585
B	14,941.3	128,352
C	16,729.1	51,131
D	15,754.7	72,453
E	16,302.6	100,397

**Table 4:** Fatigue–healing–fatigue results of different asphalt concretes.

**Type of Mix**		A	B	C	D	E
**Initial stiffness modulus of First fatigue E_0_ (MPa)**		17,513	14,941	16,729	15,755	16,303
**Initial modulus healing index HE_i_ (%)**	**HE_1_**	99.5	99.0	99.1	97.7	93.7
	**HE_2_**	99.1	96.7	97.9	96.2	92.2
	**HE_3_**	103.3	103.4	99.8	96.9	97.3
	**HE_4_**	103.2	103.8	99.7	97.9	98.6
	**HE_5_**	107.8	109.4	104.5	100.6	101.5
**Fatigue damage rate *k* (MPa/cycles)**	**k_0_**	0.072	0.035	0.102	0.099	0.060
	**k_1_**	0.285	0.132	0.429	0.291	0.138
	**k_2_**	0.436	0.338	0.550	0.382	0.209
	**k_3_**	0.349	0.082	0.323	0.152	0.177
	**k_4_**	0.389	0.071	0.335	0.213	0.186
	**k_5_**	0.443	0.101	0.364	0.143	0.198
**Measured total fatigue life N (cycles)**		185,522	405,516	141,311	238,323	313,495
**Predicted total fatigue life N_p_ (cycles)**		240,148	562,363	190,906	271,222	365,687
**Predicting fatigue life deviation (%)**		29.4	38.7	35.1	13.8	16.6

## Data Availability

The original contributions presented in this study are included in the article. Further inquiries can be directed to the corresponding author.
